# The impact of COVID-19 on private healthcare service utilisation: time series analysis in the capital region of Finland during 2020–2022

**DOI:** 10.1186/s12889-024-20594-7

**Published:** 2024-11-09

**Authors:** Oskar Niemenoja, Antti-Jussi Ämmälä, Sari Riihijärvi, Paul Lillrank, Petri Bono, Simo Taimela

**Affiliations:** 1 Aalto University, Espoo, Finland; 2 Terveystalo Plc, Helsinki, Finland; 3 University of Helsinki, Helsinki, Finland

**Keywords:** COVID-19, Cov-SARS-2, Public health, Health service utilisation, Infection epidemiology, Health policy

## Abstract

**Background:**

Most current studies on COVID-19 have focused on the first weeks or months of the pandemic or have addressed specific diseases. The long-term effects of the COVID-19 pandemic on healthcare services are insufficiently understood. We analysed the long-term effects of the COVID-19 pandemic on outpatient healthcare utilisation in the full spectrum of diseases in Uusimaa, the capital region of Finland.

**Methods:**

Our data included 632 466 individual patients between the ages of 18 and 65 and 6 521 394 visits to healthcare personnel from electronic health records. We fitted an autoregressive integrated moving average (ARIMA) model to pre-2020 data to predict the expected quantity of diagnoses for the period between 1 January 2020 and 16 June 2022. Expected and observed quantities of diagnoses were compared.

**Results:**

The overall quantity of diagnoses declined by one-fourth immediately following the onset of the pandemic and remained around 15% below predicted values for most of the pandemic. After the pandemic-related restrictions were lifted, the total diagnostic activity started to recover to pre-pandemic projection levels. However, this recovery has been mainly driven by upper respiratory system-related activity. The number of diagnoses for many diagnosis groups has remained below the predicted levels, even after the concurrent removal of mobility restrictions and increased coverage of vaccinations in this population.

**Conclusions:**

The pandemic resulted in an overall reduction in outpatient healthcare utilisation which persisted for 30 months. While the overall diagnostic activity has eventually recovered to predicted levels, many classes of diagnoses display reduced levels in the study population over the follow-up period. Some diseases that may have long-lasting effects when left untreated seem to remain underdiagnosed, potentially increasing pressure on the healthcare system in the future.

**Supplementary Information:**

The online version contains supplementary material available at 10.1186/s12889-024-20594-7.

## Background

Recent studies have shown that COVID-19 (SARS‑CoV‑2) has profoundly impacted the healthcare system beyond its direct effects. During the initial phase of the COVID-19 pandemic in May 2020, a decrease in healthcare service utilisation by about a third has been reported [1, 2]. Previous studies on healthcare utilisation during epidemics such as SARS and Ebola have demonstrated radical reductions persisting for a long time [3, 4]. Most current studies on COVID-19 have focused on the immediate first weeks or months of the pandemic or have been limited to a focused medical area, such as oncology or child health services [5, 6]. Observations of the long-term effects of COVID-19 on wider health service utilisation have been lacking. Specifically, healthcare utilisation dynamics during the later phases of the pandemic, during the easing of mobility restrictions and increases in national vaccination coverage, have not been described.

This study aims to fill this research gap by analysing trends in health service utilisation for outpatient services spanning 30 months over the course of the COVID-19 pandemic. We used extensive and novel electronic health register data from the largest private healthcare service provider in Finland, covering approximately 15% of all nationwide physician visits annually. The healthcare system in Finland is divided into three branches: public (publicly funded, publicly or privately provided), private (privately funded, privately provided), and occupational healthcare, which covers all employees (employer-funded, privately provided). The population in the study mainly consisted of occupational healthcare and private healthcare customers with a limited number of public outsourcing patients. The area of focus was the Finnish capital region of Uusimaa, which entails the greater Helsinki area and neighbouring municipalities with a combined population of 1.7 million, 31% of the Finnish population.

In Finland, the first case of COVID-19 was diagnosed in February 2020. On 18 March 2020, the Finnish Government declared a state of emergency over the coronavirus outbreak, lasting until 15 June 2020. Following the third wave of COVID-19 infections, another state of emergency was issued a year later, between 1 March 2021 and 27 April 2021, coinciding with more aggressive virus variants and slower-than-expected distribution of vaccinations. With improving vaccination coverage, most restrictions were gradually lifted towards the end of 2021. The national recommendation about remote working was lifted on 15 October 2021. To date, August 2023, there have been 1 483 607 confirmed cases of COVID-19 in Finland, of which 565 956 (38.1%) occurred within the capital region of Uusimaa [7]. At the time of writing, 84.9% of the Finnish population was vaccinated with at least two doses [8].

We evaluated the temporal variation in different diagnosis groups for outpatient care between 1 January 2020 and 27 June 2022. To estimate the effect of the pandemic on service utilisation, the post-pandemic number of weekly visits was compared with expected values reconstructed from data between 2017 and 2019.

## Methods

### Data extraction

We extracted the weekly quantity of diagnoses by ICD-10 codes, separated by year and week, from retrospective electronic health record (EHR) data. The data set included 632 466 individual patients and 6 521 394 completed diagnoses.

We included all visits from patients between the ages of 18 and 65 inside the capital region of Uusimaa between the between 1 January 2017 and 27 June 2022. Uusimaa was the region most affected by COVID-19 in Finland during 2020 and 2021 and the only area which was quarantined as traffic from and to the rest of the country was restricted during the early pandemic. Due to the occupational healthcare emphasis of the Finnish private healthcare system, only the working-age population was considered. Over 99% of the visits were outpatient appointments.

Data describing the status of the pandemic in Finland, including the number of confirmed cases, deaths, hospital bed usage and vaccinations, were obtained from the open data services provided by the Finnish Institute for Health and Welfare [9]. The numbers of confirmed cases and vaccinations were reported regionally and so represent the same geographical region as the study population, while the number of deaths and hospital bed usage represent national data, as regional data had not been made public at the time of writing this article.

### Data processing

We used the ICD-10 hierarchy level 0 as provided by the Finnish Institute of Health and Welfare, separating the data set into 22 diagnosis groups [10]. COVID-19 (U07.1, U07.2) was considered a separate group. In addition, we split the hierarchical group for respiratory system-related diseases into two, J00-J39 for acute respiratory system diseases and J40-J99 for other diseases related to the respiratory system. Especially during the early pandemic, acute respiratory system symptoms were virtually indistinguishable from those of COVID-19 without laboratory diagnostics.

To limit variance and enforce anonymity in small populations, we only considered groups where the average weekly incidence was higher than 100 diagnoses per week or 5200 per year. This limited the effects of excessive within-group variance on the results. This excluded groups D50-D89, O00-O99, P00-P96, Q00-Q99, and V01-Y98. Additionally, except for COVID-19 related codes (U07.1, U07.2), we removed the categories U00-U99, “Codes for special purposes”, and Z00-ZBB, “Factors influencing health status and contact with health services”, which contained temporary codes for individual usage and factors influencing health other than diseases, respectively. The sporadic nature of these categories makes trend-based predictions an unreliable proxy for service usage.

To improve predictive model generalisability, we excluded any major non-typical health events from the training data. This included the local norovirus epidemic in Finland for the first half of 2017, where we only included time series data for infectious and parasitic diseases (A00-B99) from July 2017 onward. We only considered annual full weeks within the data set, discarding the first and last week of the year if they did not contain the full seven days.

### Statistical methods

We calculated the weekly number of unique visits for each diagnosis group from the data. Descriptive statistics were calculated for the study population. The resulting time series data were divided into two groups. Time series from the beginning of the study period until the end of 2019 were used to train the baseline model, whereas the time series data from January 2020 onwards was compared to this value.

We used the autoregressive integrated moving average (ARIMA) based on the first data set from the years 2017 to 2019 to estimate the expected number of diagnoses in the hypothetical case without the effect of COVID-19. A unique model was calculated for each category group. Separate model parameters (*p*,* d*,* q*) were estimated for the models, and the quality of fit for the models was assessed with a Box-Ljung test on the residuals at *p* > 0.05. For any group that was not present before 2020, such as the number of COVID-19 cases or the national data for hospitalisations or vaccinations, these estimates were not calculated.

These expected values were compared with the observed values from the second data group from January 2020 until the end of the observation period. Where pre-2020 estimates were not available, only the observed values were considered. To compare the difference between the expected and observed service usage, we calculated the weekly absolute and cumulative differences between the expected spot values and observed values. The cumulative values were further divided by the weekly expected cumulative values to obtain the proportional value of how much below the expected service usage the observed values were. Three distinct service utilisation recovery patterns were identified from the data. Finally, we performed a visual analysis of the resulting values.

The calculations were performed using the R statistical language (version 4.0.5). For the predictions, 95% CI refers to the 2.5th and 97.5th percentiles of the presented distributions. Codes are available upon reasonable request from the authors.

## Results

The number of visits and descriptive statistics per ICD-10 diagnosis group are outlaid in Table 1. The most common diagnosis groups were respiratory system related (18% of all visits), musculoskeletal system related (16%) and mental and behavioural disorder related (9%). The fewest diagnoses were within perinatal conditions, congenital and chromosomal abnormalities, pregnancy, and blood-related diagnoses, all of which constituted less than 0.5% of all visits. The population was predominantly female (57%), 74% of the visits were occupational healthcare-related, and the most common age category of the patients was 30–39 years (25%).

**Table 1:** The descriptive statistics for the data set and each different diagnosis group. For each group, the total number of visits, individual patients, gender distribution, and age are reported. As only categorical age was collected, the age is represented as the mode of the ages in the pool. Ages were categorised as (% of all patients): < 30 (20.0%), 30–39 (24.6%), 40–49 (23.4%), 50–59 (22.8%), 60–65 (9.2%)

Diagnosis group	ICD-10	Visits, total	Persons, total	Gender (%, female)^d^	Age, mode	Highest age mode, fraction of all
Total		6 521 394	632 466	56,7%	30–39	24,6%
COVID-19	U07.1, U07.2	187 104	95 220	55,2%	30–39	28,6%
Certain infectious and parasitic diseases	A00-B99^c^	193 957	103 648	48,9%	50–59	27,3%
Neoplasms	C00-D48	144 426	65 929	60,7%	40–49	32,9%
Diseases of the blood and blood-forming organs and certain disorders involving the immune mechanism	D50-D89^a^	23 818	8 645	80,5%	40–49	32,9%
Endocrine, nutritional and metabolic diseases	E00-E90	129 269	50 547	54,3%	50–59	35,2%
Mental and behavioural disorders	F00-F99	561 819	130 479	62,1%	30–39	30,3%
Diseases of the nervous system	G00-G99	165 790	64 832	63,6%	40–49	26,1%
Diseases of the eye and adnexa	H00-H59	218 381	112 013	58,4%	50–59	28,1%
Diseases of the ear and mastoid process	H60-H95	140 346	73 075	52,5%	40–49	24,4%
Diseases of the circulatory system	I00-I99	190 805	72 919	45,9%	50–59	39,6%
Diseases of the respiratory system, acute	J00-J39	1 103 835	303 174	57,4%	30–39	28,4%
Diseases of the respiratory system, other	J40-J99	47 974	20 206	58,5%	50–59	27,9%
Diseases of the digestive system	K00-K93	185 736	80 931	51,4%	30–39	24,6%
Diseases of the skin and subcutaneous tissue	L00-L99	306 443	142 129	54,8%	< 30	24,6%
Diseases of the musculoskeletal system and connective tissue	M00-M99	1 061 245	256 924	53,1%	50–59	29,2%
Diseases of the genitourinary system	N00-N99	293 588	130 276	83,8%	50–59	25,9%
Pregnancy, childbirth and the puerperium	O00-O99^a^	18 930	8 816	99,9%	30–39	57,0%
Certain conditions originating in the perinatal period	P00-P96^a^	123	96	63,4%	30–39	35,8%
Congenital malformations, deformations and chromosomal abnormalities	Q00-Q99^a^	5 695	3 175	55,9%	30–39	26,8%
Symptoms, signs and abnormal clinical and laboratory findings, not elsewhere classified	R00-R99	529 748	213 757	58,3%	30–39	25,9%
Injury, poisoning and certain other consequences of external causes	S00-T98	555 237	172 708	45,7%	< 30	24,0%
Codes for special purposes	U00-U99^b^	12 438	8 236	60,3%	40–49	29,3%
External causes of morbidity and mortality	V01-Y98^a^	18 644	14 667	59,1%	30–39	23,9%
Factors influencing health status and contact with health services	Z00-ZZB^b^	420 482	220 738	57,0%	30–39	26,2%

^a^Excluded from analyses (< 100 average diagnoses per week)

^b^Excluded from analyses, temporary codes and general inquiries

^c^First half of 2017 excluded due to the local norovirus epidemic

^d^Self-reported, binary

**Fig. 1:**
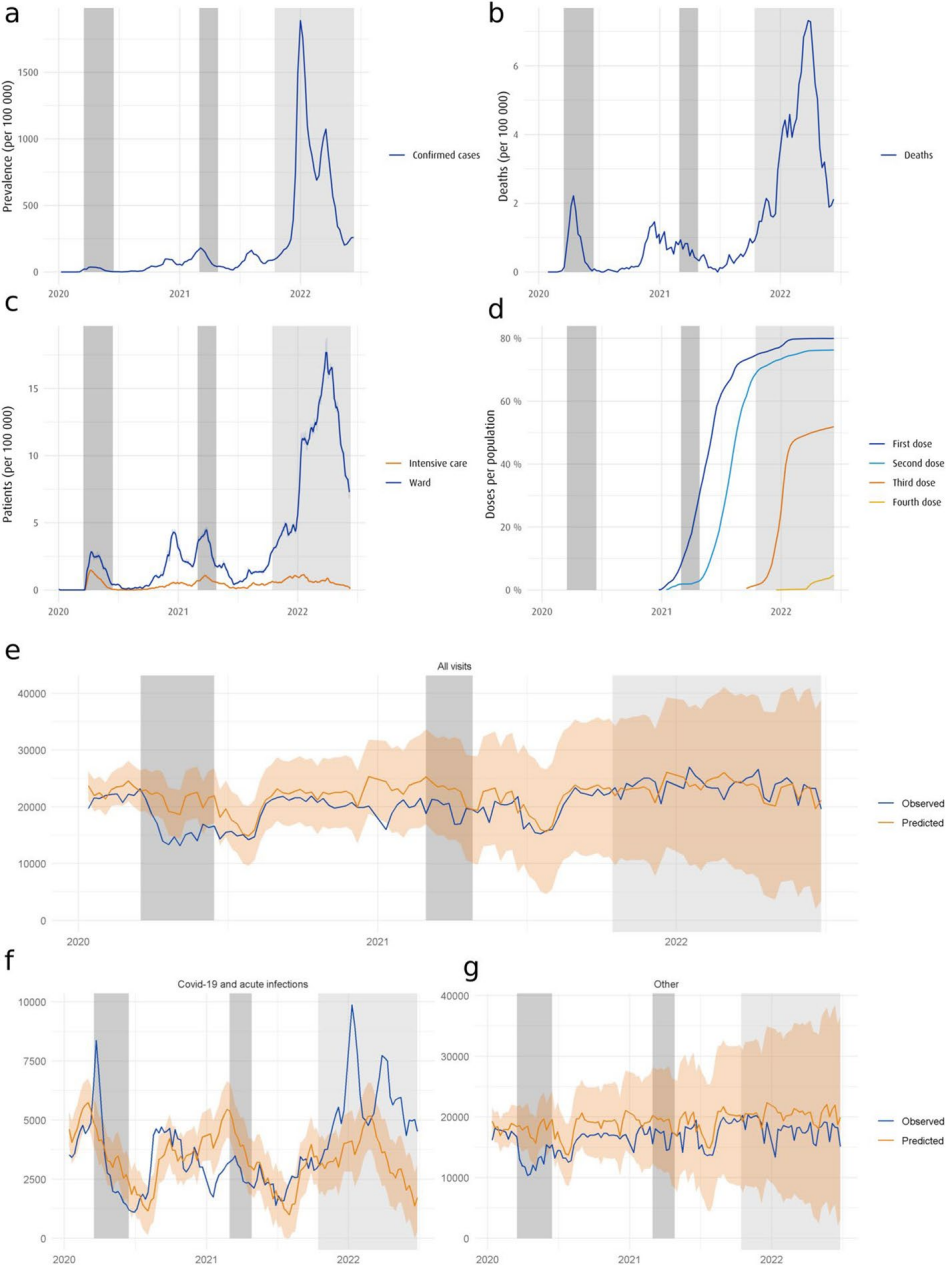
The overall COVID-19 situation in Finland and in the capital region of Uusimaa between 1 January 2020 and 16 June 2022. The grey areas represent the periods with a national state of emergency in Finland, with the lighter grey area denoting the subsequent opening of the society due to increased vaccination coverage. Subfigure (**a**) shows daily new confirmed cases of COVID-19 in Uusimaa per 100 000 inhabitants as a running 7-day average. Subfigure (**b**) shows daily new confirmed deaths from COVID-19 in Finland per 100 000 inhabitants as a running 7-day average. Subfigure (**c**) shows the cumulative number of hospitalised COVID-19 patients in Finland per 100 000 inhabitants as a 7-day running average. Subfigure (**d**) shows the percentage of the population of Uusimaa that has been vaccinated as a cumulative plot. Subfigure (**e**) shows the total weekly number of observed visits for the study population in Uusimaa. The prediction values are calculated from pre-2020 data using an ARIMA model with the ribbon denoting a 95% confidence interval. Subfigures (**f**) and (**g**) show healthcare service visits for the study population in Uusimaa stratified by diagnosis, where respiratory system-related infections, which include the ICD-10 codes for COVID-19 (U07.1, U07.2) and acute respiratory diseases (J00-J39), are separated from other visits

An overview of the COVID-19 situation in Finland between 1 January 2020 and 16 June 2022 is presented in Fig. 1. National states of emergencies and the subsequent opening of the Finnish society are shaded in grey as they coincide with the heaviest regulatory measures or the easing thereof. During the onset of the pandemic in early 2020, we can see an overall reduction in service utilisation (Fig. 1*e* and *g*), after which the values remained towards the lower end of the confidence interval until the latter half of 2021, after which the values recover to pre-pandemic projections. For acute infections (Fig. 1*f*), we observe a large initial spike in diagnostic activity followed by a persisting decrease in these visits, with a re-emergence of elevated activity levels from late 2021 until the end of the study period.

**Fig. 2:**
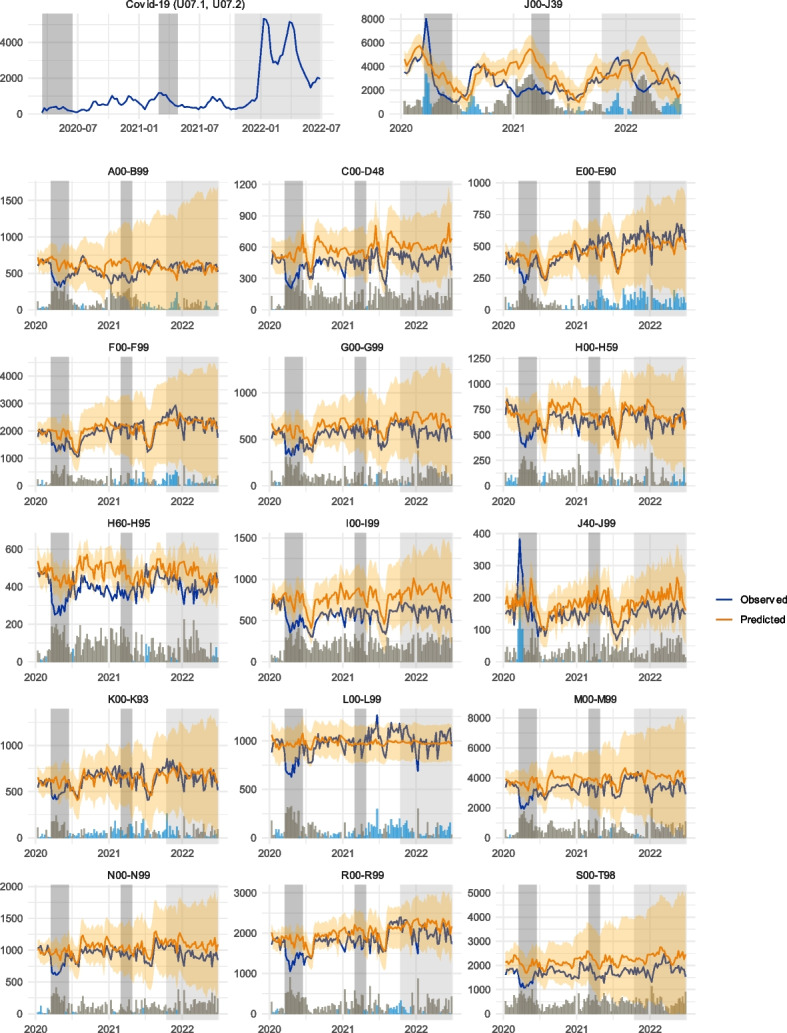
Weekly values for ARIMA-predicted and observed diagnoses for different ICD-10 diagnosis groups. The grey areas denote the periods of a state of emergency in Finland, with the lighter grey area indicating the period for reopening the society due to vaccination coverage. The bar plot shows the difference between predicted and observed values, with blue bars denoting months where the observed number of diagnoses exceeded those expected. The predicted values were calculated using an ARIMA model fitted with pre-2020 time series data. The orange ribbon represents the 95% CI of the predicted values. The full names of the ICD-10 classes are displayed in Table 1 and in the Supplementary material of this article

Figure 2 shows the number of expected and observed diagnoses by the diagnosis group. The bars below each plot denote the difference between the two values. The weeks where the observed amount was higher than the prediction have been highlighted with blue in the bar plot. The orange ribbon denotes the 95% confidence interval of the prediction model. The grey bands represent the states of emergency by the Finnish government and the subsequent gradual reopening of society due to increased vaccination rates. We can observe a marked reduction in the number of observed diagnoses during and directly following the first state of emergency for most of the diagnosis groups, coinciding with the most severe regulatory measures and initial public outreach about the pandemic in Finland. This phenomenon is less apparent during the second state of emergency, where such a decrease cannot be observed. By 2022, service utilisation rates have recovered for some diagnosis groups while others display values permanently below the predicted values. Even though the observed values remain within the 95% CI for most of the study period for most of the diagnosis groups after the first state of the emergency, they stay below the predicted spot estimates for the remainder of the study for most of the diagnosis groups.

**Fig. 3:**
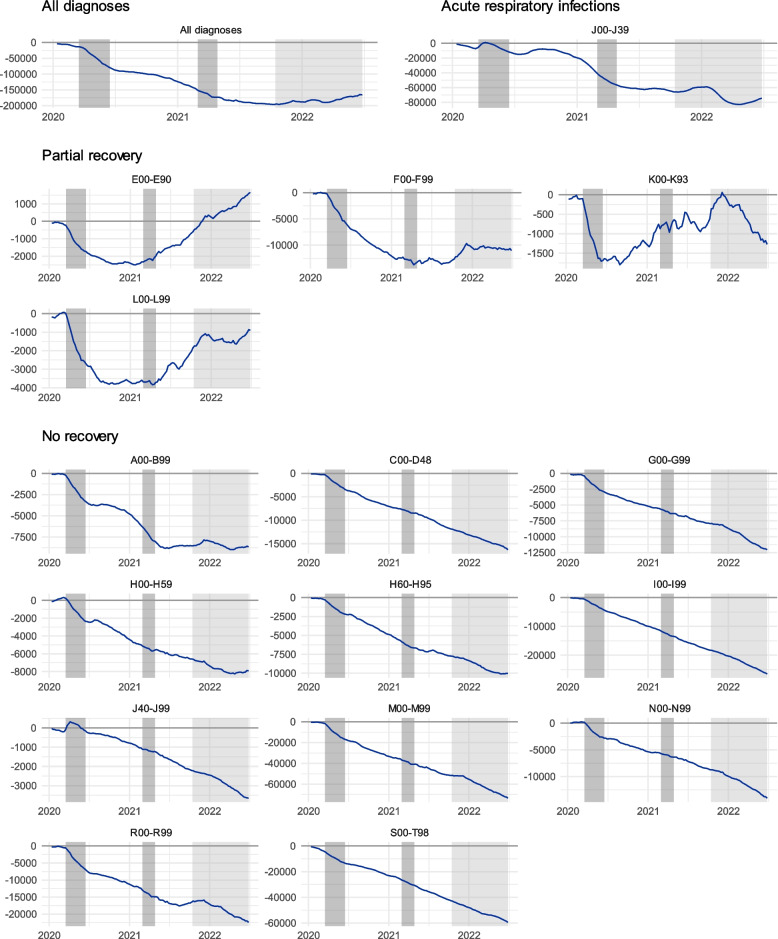
The cumulative difference in the observed and predicted number of visits by diagnosis group. The greyed areas denote the periods of a state of emergency in Finland, with the lighter grey indicating the period for reopening the society due to vaccination coverage. The values represent the absolute cumulative difference between the predicted and observed values. This difference peaks at around 200 000 visits during the observation period towards the end of 2021. The full names of the ICD-10 classes are displayed in Table 1 and in the Supplementary material of this article

**Fig. 4:**
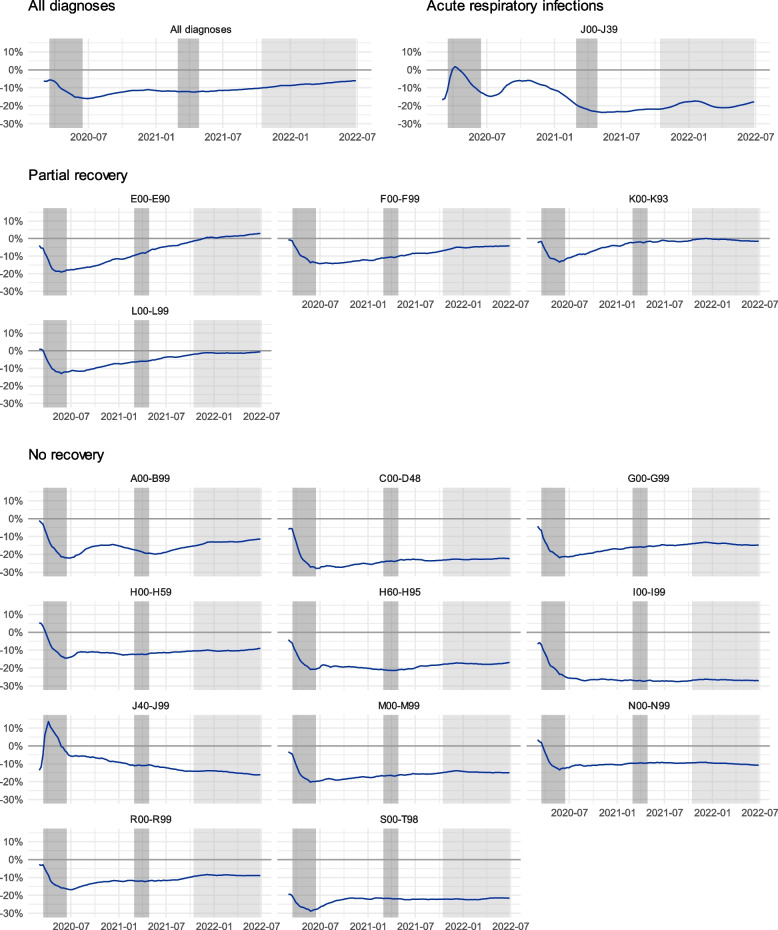
The cumulative percentage ratios of service usage. The greyed areas denote the periods of a state of emergency in Finland, with the lighter grey indicating the period for reopening the society due to vaccination coverage. The line represents the ratio of cumulative observed versus predicted values. We can observe how the partial recovery subgroup recuperates to pre-pandemic levels while the no recovery -subgroup remains below the predicted values. The full names of the ICD-10 classes are displayed in Table 1 and in the Supplementary material of this article

In general, three patterns emerge from the data. The first, communicable diseases (J00-J39 and J40-J99, see also Fig. 1), see a remarkable increase in service usage immediately following the onset of the pandemic but exhibit heavily reduced diagnosis numbers since. Secondly, some diagnosis groups exhibit a rapid initial drop and a subsequent recovery phase during 2021, finally reaching around 95% or more of the pre-pandemic visit predictions. These include mental health (F00-F99), endocrine, nutritional and metabolic diseases (E00-E99), digestive system (K00-K99) and skin and subcutaneous tissue (L00-L99) related diseases. We call this group the *partial recovery* group. Third, the remaining diagnosis groups remain constantly below the predicted service utilisation levels, with the ratio between the predicted and observed service usage stabilising 10–20% below predicted values. We call this the *no recovery* group. This group contains multiple large disease categories, such as diseases of the musculoskeletal system (M00-M99), injuries (S00-T98), cardiovascular diseases (I00-I99), and symptoms and findings not elsewhere classified (R00-R99). Figures 3 and 4 depict the cumulative differences between the predicted and observed values and describe this phenomenon. Figure 3 describes the absolute cumulative difference between the predicted and observed values, which peaked at nearly 200,000 less than predicted, with a slight trend for recuperation beginning during the first half of 2022. By the end of the observation period, the difference between the observed and predicted visits was roughly 160 000.

Figure 4 describes these cumulative percentage values of the ratio between the observed and predicted values. These represent the proportional difference between observed and predicted diagnoses at any given point. We can observe how the *partial recovery* subgroup experiences recovery after the initial drop, at times even surpassing the predicted values. The *no recovery* subgroups display a stable cumulative difference of 10–30% less than predicted, unaffected by the increase in vaccination levels towards the end of the pandemic. Groupwise values for the different states of emergencies and intermittent periods are presented in supplementary material of this article.

## Discussion

For the studied private healthcare provider in Uusimaa, throughout the COVID-19 pandemic, there was a notable decrease in the number of observed diagnoses compared to expected diagnoses for all diagnosis groups, in line with previous studies [11–15]. During the initial pandemic, the observed diagnosis activity was, on average, 24% lower than expected but varied heavily between different diagnosis groups. This period of rapid disengagement with health services was followed by a period of recovery, where the average difference between the diagnostic activity recovered to a level roughly 15% below the predicted projections. By late 2021, society entered the vaccination phase of the pandemic, where overall diagnostic activity started to recover to pre-pandemic projections. However, this change was mainly driven by an increase in acute respiratory system-related visits. Most other diagnosis groups remained considerably below their predicted values until the end of the study period.

We observe three distinct subcategories for the diagnosis groups: first, *COVID-19 and respiratory system-related diagnoses*, which are most likely to be directly associated with COVID-19 and subsequent restrictive measures, such as improved hygiene and increased attention to upper respiratory tract infections; secondly, the *partial recovery* subgroup, which seems to return to near the expected levels of service usage towards the end of 2021 and increasing vaccination coverage; and third, the *no recovery* subgroup which remains below their pre-pandemic projections for diagnostic activity. The *no recovery* subgroup remains 20–30% below their projected diagnostic activity even after the reopening of society. Previous studies have described how service usage is reduced during COVID-19 and past pandemics [1–4, 16], but few have categorised these by their recovery mechanisms.

It is unclear if the changes in service utilisation rates for the diagnosis classes, particularly for the *no recovery* group, are permanent. If they were, decreased service usage translates to excess healthcare capacity in the healthcare system, which can theoretically be reallocated to the treatment of various other diseases. A key control mechanism for the Finnish pandemic response, until sufficient vaccination coverage was reached, was intentionally delaying non-acute care to reserve healthcare resources for pandemic management, tracing, and COVID-19-related complications [17]. Future pandemics may therefore bring both opportunities and challenges for the reallocation of healthcare resources during the different phases of the pandemic.

Little attention has been given to the healthcare debt or underdiagnosis of non-COVID-related diseases during COVID-19. In total, the difference between the predicted and observed diagnoses peaked at nearly 200 000 fewer visits than predicted. The largest single diagnosis groups are diseases related to the musculoskeletal system (M00-M99) and injuries (S00-T98), which saw a combined reduction of 130 000 visits over the study period. The long-term effects depend on the nature of the diseases.

Any change in service utilisation can be seen as a result of a change in population morbidity, population behaviour or healthcare supply, or a combination of these three. Patients can also transition between service providers and from public to private and vice versa. From a time series analysis, these phenomena cannot be distinguished, and the results do not directly translate into analyses of the portion of acute or necessary care that was not delivered during the period. The decrease in diagnoses may have occurred at health check-ups or less severe symptoms and possibly among people with less severe illness [1]. In those cases, a decrease in diagnoses does not pose a corresponding long-term risk as in more severe diseases. However, delaying the diagnosis of more severe conditions, such as circulatory system-related diseases or cancer, may pose severe public health implications that persist long after the pandemic.

### Comparison with related studies

The results are in line with other studies exploring the effects of pandemics on the healthcare system for past pandemics [1, 3, 4]. Recent studies have shown similar reductions in service usage in Finland for diabetes, cancer and emergency care patients during COVID-19 [11–13]. Our analyses show that the change in healthcare utilisation was rather systematic and persisted for a long period after the onset of the pandemic.

Recent studies have shown that during COVID-19, service usage shifted rapidly to digital services [12, 18–21]. While our study did not account for the service channel, the *no recovery* group contains many conditions which may require a physical examination. For example, mental health symptoms can be treated to a higher degree via digital channels than musculoskeletal system-related diseases. This non-substitutability may be a contributing factor for the lower service utilisation rates compared to the other disease groups.

Recent studies have emphasised the need for high-quality time series data on the effects of epidemics on the health service system [4]. Our study represents a comprehensive, long-term, and representative analysis of the impact of COVID-19 between January 2020 and June 2022.

### Strengths and limitations

We had access to a large and comprehensive health register over a long period of the COVID-19 pandemic, comprising a homogenous greater capital region population. We also had access to pre-COVID-19 data for the same population, enabling us to create a credible estimate with seasonal and yearly trends for the nominal usage of health services without the impact of the COVID-19 pandemic. Compared with conventional year-on-year analysis, our results account for seasonal variances and annual trends in service usage. The results are in line with those reported previously and offer a comprehensive comparison of the various diagnosis groups.

The study cohort is more likely to have higher education and better socioeconomic status and, subsequently, lower morbidity and better access to care than the general population, and thus might not be representative of general population [22]. The data is sourced from a single private health service provider, which may create biases in the data. Register data may contain errors or omissions, but these are unlikely to cause considerable implications in the findings.

The statistical prediction model only considers historical data and is not necessarily an accurate representation of reality, especially when extrapolated over extended periods. Long-term projections for predictive data suffer from loss of accuracy during longer predictive trajectories. While the long-term nature of the study was a strength, care should be employed when analysing the later periods in the analysis.

### Implications of the findings

Service providers must be prepared and able to react to rapid changes in care demand with considerable differences between different diagnosis groups. This creates significant pressure on healthcare personnel allocation efforts during the different phases of the pandemic. Restrictive measures may have restricted the spread of infectious diseases beyond COVID-19 alone over the course of the pandemic, as indicated by the decrease in communicable diseases observed in this study. The increased vaccination rates coincide with the recuperation of health service usage. Different diagnosis classes exhibit different recovery trends, with several diagnosis classes displaying permanently reduced usage levels. These may pose long-term effects on the healthcare system if not accounted for.

Some of these shifts in service usage may be positive. The restrictive pandemic measures have likely had a positive influence on the prevalence of seasonal influenza and related diseases. During the pandemic, acute respiratory system-related diseases (J00-J39) alone have been reduced by tens of thousands of visits. This represents potential healthcare capacity that could more efficiently be reallocated if good hygiene habits continue after the pandemic. Increased public hygiene measures may positively affect the larger population in the future with decreased cases, severe cases, and sick leaves. If the changes in care utilisation have occurred in low-value services, such as overdiagnosis and treatment of relatively mild conditions, it may be more beneficial for populations and their health systems if utilisation rates of some services do not return to pre-pandemic levels [23].

## Conclusions

The pandemic resulted in an overall reduction in health service diagnoses that has persisted for an extended period. Reduction in utilisation of low-value healthcare services may have taken place, but some diseases that have long-lasting effects when left untreated have possibly been neglected. This poses an additional risk to healthcare systems beyond the direct impact of COVID-19. Different patient groups may require increased attention after the recovery phase of the pandemic to limit the possible long-term impact on the healthcare system. The effect of the reduction in health service usage should be closely monitored, and timely and necessary care for patients should be ensured during any future pandemics.

## Supplementary Information

Supplementary Material 1.

## Data Availability

Access to the data can be applied through the Finnish Social and Health Data Permit Authority Findata. The codes are available from the author upon reasonable request.
