# Supplementary material for: The impact of COVID-19 on private healthcare service utilisation: time series analysis in the capital region of Finland during 2020–2022
Source: BMC Public Health. 2024 Nov 9;24:3100. doi: 10.1186/s12889-024-20594-7 (PMC11549829; doi:10.1186/s12889-024-20594-7)
Supplement: Supplementary file 1 — Supplementary Material 1. [file 12889_2024_20594_MOESM1_ESM.zip › supplementary_material.pdf]

Table A

Changes in diagnosis-wise usages over the six different periods. The percentage values denote how much the number of observed values was below the estimates based on the pre-pandemic data. Period 1 represents the time before the onset of COVID-19, periods 2 and 4, which are highlighted in grey, denote the first and second states of emergency (SoE) in Finland; the third period indicates the time between these, the fifth period after the second state of emergency and the sixth represents the recovery phase after improved vaccination status. Total denotes the overall difference between the observed and predicted values over the whole study period, for which also absolute amounts are presented.

Group	1.1.2020 – 16.3.2020	State of Emergency 17.3.2020 – 15.6.2020	16.6.2020 – 28.2.2021	State of Emergency 1.3.2021 – 27.4.2021	28.4.2021 – 30.9.2021	Recovery phase 1.10.2021 – 16.6.2022	Total	Total, visits
A00–B99	−3,10 %	−37,20 %	−13,70 %	−32,20 %	−3,90 %	−0,60 %	−11,40 %	-8 700
C00–D48	−5,70 %	−43,80 %	−21,70 %	−19,30 %	−23,00 %	−20,00 %	−20,40 %	-17 000
E00–E90	−5,50 %	−27,10 %	−4,30 %	7,70 %	13,00 %	13,00 %	12,60 %	1 200
F00–F99	−1,20 %	−23,50 %	−9,20 %	−5,10 %	0,00 %	2,00 %	2,00 %	-11 000
G00–G99	−6,60 %	−32,50 %	−12,60 %	−13,00 %	−10,00 %	−15,00 %	−16,40 %	-12 300
H00–H59	3,40 %	−29,10 %	−10,70 %	−10,00 %	−6,00 %	−5,00 %	−5,50 %	-7 950
H60–H95	−6,00 %	−33,10 %	−20,80 %	−23,30 %	−10,00 %	−13,00 %	−14,00 %	-10 000
I00–I99	−7,00 %	−38,60 %	−28,00 %	−29,40 %	−27,00 %	−27,00 %	−27,20 %	-26 500
J00–J39	−11,50 %	−13,80 %	−23,10 %	−51,10 %	−15,00 %	−8,00 %	−7,40 %	-72 000
J40–J99	−4,80 %	−2,60 %	−16,30 %	−10,70 %	−25,00 %	−20,00 %	−20,40 %	-4 100
K00–K93	−1,60 %	−20,00 %	4,40 %	1,10 %	0,00 %	−2,00 %	−2,70 %	-1 200
L00–L99	0,10 %	−21,30 %	−2,60 %	−1,50 %	7,00 %	3,00 %	2,80 %	-900
M00–M99	−4,50 %	−31,70 %	−14,40 %	−15,20 %	−11,00 %	−15,00 %	−15,00 %	-72 000
N00–N99	1,90 %	−23,80 %	−7,90 %	−8,80 %	−9,00 %	−13,00 %	−13,90 %	-13 500
R00–R99	−2,90 %	−26,80 %	−8,50 %	−13,70 %	−5,00 %	−7,00 %	−7,70 %	-22 500
S00–T98	−20,30 %	−34,40 %	−17,30 %	−24,00 %	−23,00 %	−20,00 %	−20,30 %	-59 000
All	−6,60 %	−25,30 %	−11,20 %	−17,00 %	−7,00 %	1,00 %	1,40 %	-160 000

Table B

The ICD-10 main level groups and their names.

ICD-10 code	Group
A00–B99	Certain infectious and parasitic diseases
C00–D48	Malignant neoplasms
D50–D89	Diseases of the blood and blood-forming organs and certain disorders involving the immune mechanism
E00–E90	Endocrine, nutritional and metabolic diseases
F00–F99	Mental and behavioural disorders
G00–G99	Diseases of the nervous system
H00–H59	Diseases of the eye and adnexa
H60–H95	Diseases of the ear and mastoid process
I00–I99	Diseases of the circulatory system
J00–J99	Diseases of the respiratory system
K00–K93	Diseases of the digestive system
L00–L99	Diseases of the skin and subcutaneous tissue
M00–M99	Diseases of the musculoskeletal system and connective tissue
N00–N99	Diseases of the genitourinary system
O00–O99	Pregnancy, childbirth and the puerperium
P00–P96	Certain conditions originating in the perinatal period
Q00–Q99	Congenital malformations, deformations and chromosomal abnormalities
R00–R99	Symptoms, signs and abnormal clinical and laboratory findings, not elsewhere classified
S00–T98	Injury, poisoning and certain other consequences of external causes
U00–U99	Codes for special purposes
V01–Y98	External causes of morbidity and mortality
Z00–ZZB	Factors influencing health status and contact with health services
